# Liver Transplantation for Treatment of Unresectable Spontaneous Ruptured Hepatocellular Adenoma: A Rare Indication

**DOI:** 10.7759/cureus.27788

**Published:** 2022-08-08

**Authors:** Patrícia Rocha, Nuno Leal, Manuel Barbosa, Janine De Resende, Pedro Rodrigues

**Affiliations:** 1 Internal Medicine, Centro Hospitalar do Médio Ave EPE, Santo Tirso, PRT; 2 Internal Medicine, Centro Hospitalar de Vila Nova de Gaia/Espinho EPE, Vila Nova de Gaia, PRT

**Keywords:** liver transplantation, transarterial embolization, haemorrhage, adenoma rupture, hepatocellular adenoma

## Abstract

Hepatocellular Adenomas (HA) are rare benign tumors of the liver which occur predominantly in young women. Although benign, HA may have complications such as hemorrhage and malignant transformation. So, sometimes conservative management is not enough.

We report a case of a 26-year-old woman on oral contraceptives who presented with acute abdominal pain and signs of hemodynamic shock. She underwent transarterial embolization due to the presence of multiple HA with rupture and active hemorrhage. This minimally invasive treatment failed to control the disease. The patient presented a progressive increase in the size of the masses with signs of recent hemorrhage, and the HA became unresectable, so she underwent liver transplantation.

Liver transplantation is rarely indicated for the treatment of HA; however, in unresectable masses, it should be considered to prevent potential rupture with hemorrhage or malignant transformation.

## Introduction

Hepatocellular Adenoma (HA) are benign liver tumor with an incidence of 1-4 per 1000,000 adults per year [1]. They are most common in women of reproductive age, and the incidence is 30 times higher in oral contraceptive users [2,3].

Although they are benign lesions, HA can undergo two life-threatening complications. Rupture and subsequent hemorrhage is a complication found in 15%-40% of patients with HA, and malignant transformation into hepatocellular carcinomas is found in 5% of those tumors. The risk of both complications is higher with increasing tumor size [2-7].

Treatment strategies for HA range from conservative measures with oral contraceptive discontinuation and radiologic surveillance to liver resection or transplantation [5-11].

Liver transplantation for HA has been reported but is a rare indication. So far, a few case reports were described, a large multicenter study from Europe reported 49 cases of liver transplantation for liver adenomatosis between 1986 and 2013, and a systematic review in the United States reported 142 cases of transplantation for HA between 1987 and 2020 [9-14].

We report a case of a young woman with ruptured hepatocellular adenoma with hemorrhage, who underwent liver transplantation after failure of disease control with transarterial embolization (TAE).

## Case presentation

A 26-year-old woman was admitted to the emergency department complaining of abdominal pain for two hours in February 2021. Her medical history included obesity, diabetes mellitus, arterial hypertension, autoimmune thyroiditis, depressive disorder, and oral contraceptive use.

**Figure 1:**
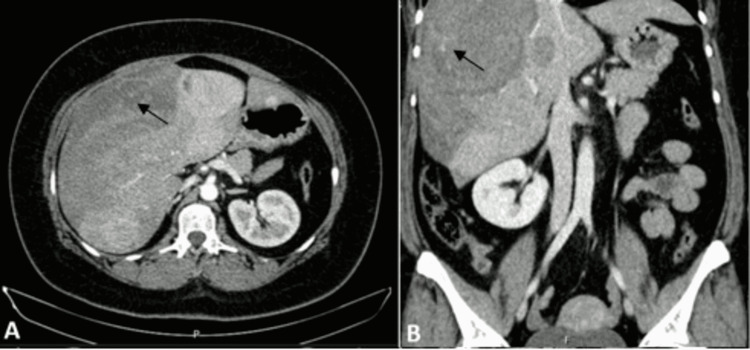
Computed tomography (CT) of the abdomen on the day of presentation. Axial (A) and coronal (B) contrast-enhanced CT images demonstrated heterogeneously hyper-enhancing hepatic masses. The largest was one in the right lobe, which measured 11 cm and one in the left lobe, which measured 6.7 cm. The CT also showed a right subcapsular haematoma with signs of active hemorrhage (arrows) measuring 22x16x10 cm.

On physical examination, the patient was afebrile, with an initial blood pressure of 130/78 mmHg, heart rate of 68 beats per minute, and significant tenderness to palpation over the right upper quadrant. The rest of the examination findings were unremarkable. The initial laboratory results showed a hemoglobin of 14.5 g/dl, alanine aminotransferase (ALT) of 75 U/L, and aspartate aminotransferase (AST) of 66 U/L. A contrast-enhanced computed tomography (CT) of the abdomen demonstrated a right subcapsular hematoma, measuring 22x16x10 cm, with active hemorrhage and various HA (the largest being on the right lobe with 11 cm and one in the left lobe with 6.5 cm) (Figures 1A, 1B). 

**Figure 2:**
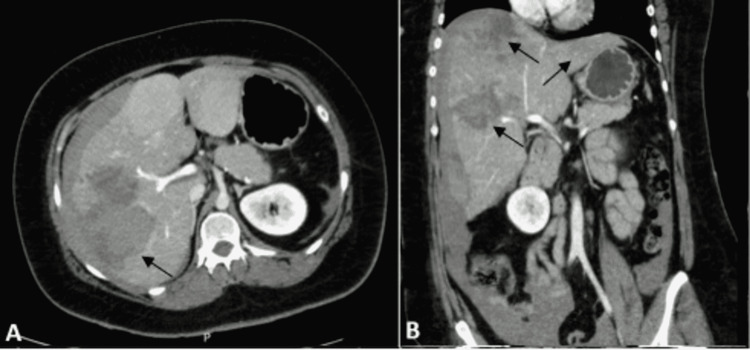
Computed tomography (CT) of the abdomen hours after first transarterial embolization. Axial (A) and coronal (B) CT angiography images demonstrated an increase to 13 cm of the mass in the right lobe, with signs of active hemorrhage and intraperitoneal effusion. Also, the CT showed two other hyperenhancing masses in the right lobe (arrows), various masses in the left lobe (arrows), the largest measuring 9.1 cm, and the right subcapsular hematoma, without signs of active hemorrhage.

A repeated blood test showed a drop in hemoglobin level to 9.5 g/dl, so she was transferred to another hospital for interventional radiology. She underwent selective TAE on suspected arteries providing a vascular supply to three hypervascular masses in the right lobe of the liver. A few hours later, she became disoriented, her blood pressure was 134/81mmHg, her heart rate raised to 130 beats per minute, and she had a hyperlactatemia of 16 mmol/L and hemoglobin of 7.7g/dl. A repeat CT scan showed an increased size of adenomas, signs of active hemorrhage of one hepatic mass in the right lobe, and intraperitoneal effusion (Figures 2A, 2B). 

A diagnosis of hemorrhagic shock was made, so she received a blood transfusion and underwent a second selective TAE of the vessels that supplied the hypervascular mass in the right lobe.

**Figure 3:**
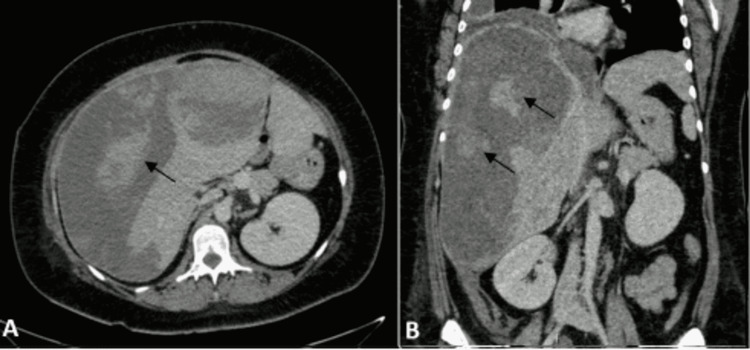
Computed tomography (CT) of the abdomen twenty-four days after second transarterial embolization. Axial (A) and coronal (B) CT angiography images demonstrated various heterogeneously hepatic masses with signs of recent hemorrhage, and increased measures (arrows). The largest were two in the right lobe, which measured 14 and 11 cm and one in the left lobe, which measured 10 cm. Also, the right subcapsular hematoma showed signs of recent hemorrhage and increased measures to 27x20x12cm.

After the second TAE, she remained in the hospital. During hospitalization, the oral contraceptive pills were discontinued. The patient underwent surveillance of the adenomas by serial CT scans that showed a progressive increase in the dimensions of the masses, without active hemorrhage but with signs of recent hemorrhage. Twenty-four days after the second embolization, she presented acute abdominal pain, hypotension of 88/50 mmHg, tachycardia of 150 beats per minute, and hemoglobin drop from 8.9 g/dl to 6.4 g/dl. A CT scan showed an increase in the size of HA and subcapsular hematoma, with signs of recent hemorrhage (Figures 3A, 3B). 

She received a blood transfusion and became hemodynamically stable. Considering the lack of clinical stability, the unresectable liver lesions due to insufficient remaining liver volume, and the high risk of rupture and hemorrhage of the lesions, the patient was proposed and approved for liver transplantation. The patient was transferred to a referral center for liver transplantation. Laboratory evaluation showed abnormal liver tests, including an increased ALT of 106 U/L, AST 53 U/L, alkaline phosphatase 579 U/L, gamma-glutamyltransferase 515 U/L, prothrombin time of 19 seconds, and a decreased albumin of 2.7 g/dl. The α-fetoprotein was normal.

In April 2021, she underwent deceased donor liver transplantation with the piggyback technique. The explant liver weighed 4050 g, and there was no evidence of tumor invasion into the capsule or blood vessels. The major part of the tumor mass was hemorrhagic and necrotic. Based on the molecular classification, the final pathologic diagnosis was exon 7-8 β catenin activated HA and unclassified HA.

A follow-up two months after the surgery revealed normal liver tests, and the CT scan showed no sign of recurrence of tumor or metastasis. She is well one year after liver transplantation, immunosuppressed with tacrolimus and mycophenolate mofetil.

## Discussion

HA are rare benign liver tumors. Its incidence has risen in the last decades due to the increased use of oral contraceptives, the prevalence of obesity and metabolic syndrome, and the diffuse use of imaging radiology with its incidental discovery [10,14,15]. These tumors occur predominantly in women of reproductive age, with a female:male ratio of 10:1. The use of anabolic androgenic steroids, some genetic syndromes like glycogen storage diseases, and some environmental factors such as obesity are also associated with the development of HA [5-7,15]. Our patient was a young obese woman with oral contraceptive use.

Based on the molecular behavior, HA has been classified into five main groups: hepatocyte nuclear factor 1α-mutated HA, inflammatory HA, exon 3 β-catenin-mutated HA, exon 7-8 β-catenin mutated HA, and unclassified HA. In 2017 Jean-Charles Nault et al. proposed a new classification with three more subtypes. It is known that β-catenin-mutated HA are at increased risk for hemorrhage and inflammatory HA are at increased risk for hemorrhage and malignant transformation, so this classification can be useful to decide the best treatment option for each patient. However, although magnetic resonance imaging is the modality of choice for the diagnosis of HA, it does not allow to reach an accurate differentiation between subtypes [3,6,7,15,16].

HA is typically asymptomatic and incidentally found on physical examination, when abdominal imaging is performed for other reasons, or during exploratory laparotomy for another indication. The remaining patients are symptomatic and could have abdominal pain due to hemorrhage within the tumor or acute intraperitoneal hemorrhage due to rupture of the tumor. Rarely HA can undergo malignant transformation [5-10]. Our patient presented initially with sudden abdominal pain associated with hemorrhage within the tumors and then had a period of hemodynamic instability due to rupture of the tumor with intraperitoneal effusion.

The management of HA can vary according to patient and tumor characteristics. All women should discontinue oral contraceptives, and the obese should lose weight. In most patients, considering the risk of bleeding or malignant transformation, conservative management with radiologic surveillance is not enough. So, surgical resection is recommended for women with tumor diameters greater than 5 cm, for all men regardless of the tumor size and if there is a proven β-catetin activated HA. The optimal treatment for ruptured HA is not established. However, considering the high risk of morbidity and mortality with emergent surgery, TAE should be considered to stabilize the patient, and the liver resection should be delayed to an elective procedure. Also, some authors have suggested that TAE can reduce or even lead to the disappearance of some tumors [1-2,4-8,10,15].

If resection is not technically feasible or brings high operative morbidity or mortality, liver transplantation should be considered. In rare cases, it should also be considered for patients with ruptured HA when conservative measures fail, and surgical resection is not feasible [9,10,13,14]. Our report represents a rare case of an unresectable spontaneous ruptured HA with a progressive increase in tumor size despite embolization, who underwent liver transplantation.

It is difficult to make evidence-based criteria for liver transplantation in HA. All cases should be discussed by a multidisciplinary group since this is a benign disease. There is high morbidity and mortality associated with transplantation, and liver grafts are scarce [12,14].

## Conclusions

HA are rare liver tumors that most commonly occur in young women with oral contraceptive use. Although benign, HA can undergo two life-threatening complications: rupture and subsequent hemorrhage and malignant transformation. These risks increase with increasing tumor size. The treatment should be individualized and can range from conservative measures to surgical resection or liver transplantation. Although liver transplantation is a rare indication, it should be considered if the resection is associated with high morbidity and mortality or is technically not feasible.
